# Gray-Scale Median in Patients with Symptomatic and Asymptomatic Carotid Atherosclerosis—Risk Factors and Diagnostic Potential

**DOI:** 10.3390/biomedicines12071594

**Published:** 2024-07-17

**Authors:** Adam Płoński, Dariusz Pawlak, Adam F. Płoński, Jerzy Głowiński, Grzegorz Madycki, Krystyna Pawlak

**Affiliations:** 1 Department of Vascular Surgery and Transplantation, Medical University of Bialystok, 15-276 Bialystok, Poland; adamplonski@op.pl (A.P.); adamfplonski@yahoo.pl (A.F.P.); jglow@wp.pl (J.G.); 2 Department of Pharmacodynamics, Medical University of Bialystok, 15-222 Bialystok, Poland; dariusz.pawlak@umb.edu.pl; 3 Department of Vascular Surgery and Angiology, Centre of Postgraduate Medical Education, Bielanski Hospital, 01-809 Warsaw, Poland; g.madycki@interia.pl; 4 Department of Monitored Pharmacotherapy, Medical University of Bialystok, 15-222 Bialystok, Poland

**Keywords:** gray-scale median, carotid plaque, carotid endarterectomy, symptomatic carotid artery stenosis, asymptomatic carotid artery stenosis, risk factors

## Abstract

Background: The identification of clinical factors affecting the gray-scale median (GSM) and determination of GSM diagnostic utility for differentiating between symptomatic and asymptomatic internal carotid artery (ICA) stenosis. Methods: This study included 45 patients with asymptomatic and 40 patients with symptomatic ICA stenosis undergoing carotid endarterectomy (CEA). Echolucency of carotid plaque was determined using computerized techniques for the GSM analysis. Study groups were compared in terms of clinical risk factors, coexisting comorbidities, and used pharmacotherapy. Results: Mean GSM values in the symptomatic group were significantly lower than in the asymptomatic group (*p* < 0.001). Both in the univariate as well as in the multiple regression analysis, GSM was significantly correlated with D-dimers and fasting plasma glucose levels and tended to correlate with β-adrenoceptor antagonist use in the symptomatic group. In asymptomatic patients, GSM was associated with the presence of grade 2 and grade 3 hypertension, and tended to correlate with the use of metformin, sulfonylureas, and statin. Independent factors for GSM in this group remained as grade 3 hypertension and statin’s therapy. The receiver operating characteristic (ROC) analysis revealed that GSM differentiated symptomatic from asymptomatic ICA stenosis with sensitivity and specificity of 73% and 80%, respectively. Conclusion: The completely diverse clinical parameters may affect GSM in symptomatic and asymptomatic patients undergoing CEA, whose clinical characteristics were similar in terms of most of the compared parameters. GSM may be a clinically useful parameter for differentiating between symptomatic and asymptomatic ICA stenosis.

## 1. Introduction

According to the data of the World Health Organization, ischemic stroke is the second cause of death and one of the main causes of permanent disability of adults in the world [1]. Carotid atherosclerosis and rupture of unstable carotid plaque is one of the main reasons of ischemic stroke [2,3]. For many years, the treatment of carotid stenosis was based on the degree of carotid stenosis and/or patient’s symptoms. Nowadays, the stability of plaque rather than the degree of arterial stenosis is recognized as a risk factor of cerebrovascular events [3]. There is also no doubt that stable atherosclerotic plaques are less susceptible to rupture than unstable plaques [4], but imaging techniques that can detect potentially unstable plaques are not routinely performed in clinical practice. A valuable tool that can be used to assess the severity of the atherosclerotic plaque and the risk of its rupture can be the gray-scale median (GSM). This computer-assisted plaque characterization is calculated on the basis of the echogenicity of the ultrasound image. The digital post-processing allows for the quantitative evaluation of differences in plaque echolucency between symptomatic and asymptomatic plaques, providing additional information on plaque morphology [5,6,7]. Thus, the GSM may be a good marker of plaque instability, with the potential clinical use due to its reliability and simplicity of assessment, ability to be calculated from plaque images collected from clinical B-mode ultrasonography, and low cost of study. Numerous studies have shown that GSM reflects the composition of plaques in patients who underwent carotid endarterectomy (CEA) [8,9,10,11,12,13]. It has also been widely accepted that high GSM values define the plaques as more echogenic, with a higher grade of calcification and fibrosis, and potentially more stable. On the contrary, low GSM values with high lipid content and a thin fibrous cap correlated with echolucent, vulnerable plaques [14,15,16,17], and the patients with features of instability of the atherosclerotic plaque found in its structure are at higher risk of cerebral ischemic episodes [6,13,18,19]. The numerous risk factors, which are linking with carotid plaque instability, have been recognized. They include uncontrollable factors, such as age, male sex, and genetic factors. The modifiable factors, like high blood glucose, dyslipidemia, hypertension, obesity and smoking, are increasingly emphasized by many authors [14,15,17,20,21]. The identification of risk factors associated with instability of carotid plaque, especially in symptomatic patients, is critical for making the proper decision with regard to lifestyle changes, pharmacological treatment, or type of interventional therapy. Therefore, the aim of this study was the identification of factors affecting GSM in symptomatic and asymptomatic patients undergoing CEA, based on data collected in routine clinical practice. The diagnostic utility of the GSM to differentiate between symptomatic and asymptomatic ICA stenosis was also assessed.

## 2. Materials and Methods

### 2.1. Participants

This study included 85 consecutive patients undergoing carotid endarterectomy (CEA) in the Department of Vascular Surgery and Transplantation, Medical University of Bialystok, between January 2021 and December 2022. In patients included in the study group, atherosclerotic plaques caused stenoses of the internal carotid arteries in the range from 60 to 99% according to NASCET in angio-CT examination [22]. General inclusion criteria for the CEA procedure were symptomatic patients with ≥50% carotid stenosis, and asymptomatic patients with ≥60% carotid stenosis, as recommended by the European Stroke Organization guideline on endarterectomy [23]. Given that patients were assessed for surgical eligibility by neurologists, the pertinent indications specific to their expertise were retained. The exclusion criteria were as follows: cerebral hemorrhage, intracranial tumors, aneurysms, heart failure, atrial fibrillation, and severe liver or kidney disorders. The patients included in this study were divided into two groups, according to the symptomatology of carotid artery stenosis. A symptomatic patient, according to the current definition [23], was a patient who had a documented ischemic episode of the central nervous system in the form of a completed ischemic stroke, or a transient ischemic episode (TIA), within the past 6 months. The group of patients defined as symptomatic consisted of 40 (47.06%) people, and the group of asymptomatic patients consisted of 45 (52.94%) people.

### 2.2. Clinical and Biochemical Data Collection

#### 2.2.1. Subjective Examination

Each patient in the research group had a detailed medical history. During the interview, information was collected on the course of the disease and its diagnosis, risk factors for atherosclerosis and comorbidities, and medications taken by the patient. Diabetes mellitus (DM) was diagnosed if fasting blood glucose was at least 126 mg/dL, or if the subject had previously diagnosed DM and used an oral hypoglycemic drug or insulin treatment [24]. Dyslipidemia was diagnosed if serum total cholesterol (TC) was >190 mg/dL, low-density lipoprotein cholesterol (LDL-C) was >115 mg/dL, high-density lipoprotein cholesterol (HDL-C) was <45 mg/dL for women and <40 mg/dL for men, triglycerides were >150 mg/dL, or lipid-lowering drugs were used [25]. Coronary artery disease (CAD) was defined as acute myocardial infarction (MI), acute coronary syndrome (ACS), or ischemic heart disease (IHD), according to the European Society of Cardiology guidelines [26]. Kidney function was assessed according to eGFR values, which were estimated at baseline for all patients before CEA, calculated using the Chronic Kidney Disease Epidemiology Collaboration (CKD-EPI) equation and expressed in mL/min/1.73 m^2^ [27]. Patients with eGFR values < 60 mL/min/1.73 m^2^ for longer than three months were classified as having chronic kidney disease (CKD), according to the National Kidney Foundation’s Kidney Disease Outcome Quality Initiative (NKF KDOQI) guidelines [28]. Patients who were using tobacco products on admission to our hospital were considered smokers. We combined the questionnaire data on smoking with packs per year values, and packs per year of smoking were computed as the average number of packs of cigarettes per day multiplied by the duration of smoking in years [29].

#### 2.2.2. Routine Biochemical Measurements

Fasting blood glucose, creatinine, D-dimers, and lipid profile were determined by standard methods (Cobas C311, Roche, Mannheim, Germany). Serum C-reactive protein (CRP) was analyzed using a nephelometric technique (Beckman Coulter Immage 800; Fullerton, CA, USA; normal range: 0–0.8 mg/dL).

#### 2.2.3. Physical Examination

All patients encompassed in this study underwent thorough consultation with neurology, vascular surgery, and radiology specialists. The ultimate decision was reached collaboratively, following comprehensive evaluation encompassing angioCT examinations of the head and neck, both with and without contrast, as well as an in-depth analysis of color Doppler ultrasound assessments, including a gray-scale analysis. We defined 75 cases of hypertension in this group (88.24%) by either elevated blood pressure values, self-reported previous diagnosis, or use of antihypertensive medications. The hypertension was categorized as grade 1 hypertension, grade 2 hypertension, and grade 3 hypertension, according to the European Society of Hypertension Guidelines [30]. During the physical examination, data on the patient’s weight and height were collected, which were then used to determine the body mass index (BMI) [31].

### 2.3. Ultrasound Examination and Carotid Plaque GSM Analysis

#### 2.3.1. Ultrasound Evaluation

One day before the procedure of carotid endarterectomy, during preparation for the surgery, each patient from the study group underwent a color Doppler ultrasound examination of the carotid arteries and atherosclerotic plaque assessment in the GSM. Each of the examined patients underwent a detailed ultrasound examination with assessment of both carotid arteries. During the Doppler ultrasound examination of the carotid arteries, the following were assessed:

(a) Atherosclerotic plaque—during the ultrasound examination, the best possible image projection in B-mode presentation was selected with the entire atherosclerotic plaque visible—the image was not enlarged or brightened—according to the accepted standardization. The image of the plaque was recorded on a photograph. Then, to assess the atherosclerotic plaque, images from the ultrasound examination were used to analyze its structure on the GSM scale.
(b) The degree of stenosis of the internal carotid artery [%]—by recording the spectrum of blood flow in the internal and the common carotid artery. The obtained velocities [m/s] of PSV (peak systolic velocity) and EDV (end diastolic velocity) flow are the basis for estimating the degree of stenosis of the internal carotid artery expressed as a percentage in the form of a range of values with a tolerance of up to 10% [32]. In our study, the degree of stenosis in all patients was assessed by calculating the ratio of peak systolic velocity measured at the stenotic site, typically the internal carotid artery, to the peak systolic velocity in the central segment of the common carotid artery. We considered the ratio of end diastolic velocity at the stenotic site to the end diastolic velocity in the midsection of the common carotid artery [32].

The record of the course of the ultrasound examination together with the measurements taken was documented in the form of images in the DICOM format, which were stored on an electronic medium. In the case of bilateral carotid artery stenoses in symptomatic patients, the atherosclerotic plaque from the internal carotid artery (ICA) that caused CNS ischemic symptoms was used for this study. In the case of bilateral stenoses in asymptomatic patients and in symptomatic patients in whom CNS ischemia manifested itself in the form of disorders undifferentiated as to the side causing them (e.g., speech disorders), the atherosclerotic plaque in ICA, which caused greater stenosis, was selected for this study.

In the DICOM format, we stored

- Images of the visible atherosclerotic plaque in the carotid artery.
- Images containing records of measured blood flow velocities in the carotid artery.

All ultrasound examinations were performed with the use of the ultrasound device and the VF13-5 linear transducer with a frequency band of 4.4–13 MHz (SIEMENS ACUSON X300, Siemens Healthcare, Erlangen, Germany). All studies were conducted by the same researcher (A.P.).

#### 2.3.2. Image Standardization

Obtained DICOM images with visible atherosclerotic plaque were formatted. Images in jpg format were enlarged 2 times and analyzed in the Liver Analyzer version 2.8.6.3c, evaluating the gray-scale median image based on Adobe Photoshop software Version 22.1.1 (Adobe Systems, San Jose, CA, USA). Using the software, the structure of atherosclerotic plaque was assessed based on its echogenicity in the ultrasound image.

#### 2.3.3. Calculation of the GSM Value of Atherosclerotic Plaques

The ultrasound device’s video signal was converted into a digital image format by a personal computer, and a subsequent analysis was conducted on images initially sized. Echogenicity was quantified using the gray-scale median (GSM) derived from the frequency distribution of gray-scale values of pixels within the entire plaque or a defined region thereof. An in-house-developed program was employed to execute the following procedures: Initially, all carotid plaques underwent automatic linear scaling after the examiner outlined a region corresponding to blood and another corresponding to the adventitia. Following normalization, the gray-scale values were set to 0 for blood and 195 for adventitia. Subsequently, the plaque was outlined in its longitudinal section to generate a binary map. The luminal margin was then outlined once more to provide the program with precise plaque surface localization. The aim of our research was not to develop a new method for analyzing ultrasound images of atherosclerotic plaques. Instead, we aimed to utilize an existing, well-researched, cost-effective, and straightforward image processing method to create a tool that enhances the accuracy of diagnosing and making intervention decisions for patients who are difficult to assess and exhibit ambiguous symptoms.

**Figure 1:**
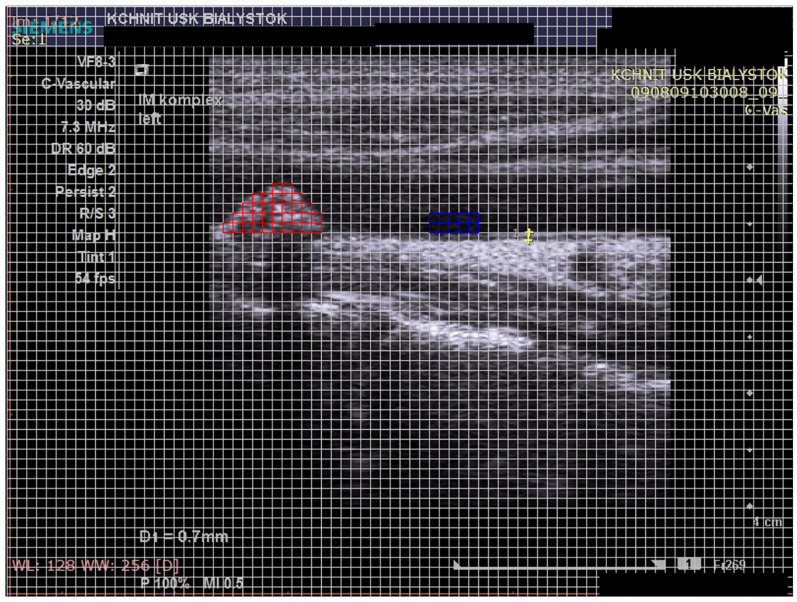
The analysis of atherosclerotic plaque on the grey-scale median (GSM). The red color is used to mark the tested object—atherosclerotic plaque. The blue color is used to mark the area, which is a reference value—blood in the lumen of the artery.

The program allows the evaluation of the tested object by imposing a matrix of an equal size of boxes on the image. Thanks to the applied matrix, it is possible to mark the entire tested object by marking individual boxes with a color within its borders. The program allows the calculation of the degree of image brightness within each of the marked boxes. The program calculates the average value (GSM1) from the absolute values obtained from the individual boxes marked in the GSM (Figure 1). 

In this study, the entire atherosclerotic plaque was marked with red, without crossing its borders. Prior to determination, the atherosclerotic plaque was outlined. The number of marked squares depended on the size of the examined plaque. The program automatically calculated the average value for the entire examined lesion. In this study, the lumen of the artery was marked with blue, selecting the area corresponding to the place with the greatest shadowing (hypoechoic), without artifacts. The lumen of the artery was marked using 10 boxes each time. A potential measurement error (resulting, for example, from a different degree of total brightness of the image) was automatically corrected by the program by referring the obtained average GSM value of the plaque (GSM1) to the average value obtained from the measurement of the lumen of the vessel filled with blood (GSM2). The obtained mean value—GSM 1 (atherosclerotic plaque)—was reduced by the mean value of GSM 2 (artery lumen) as follows:

GSM 1 − GSM 2 = GSM

**Figure 2:**
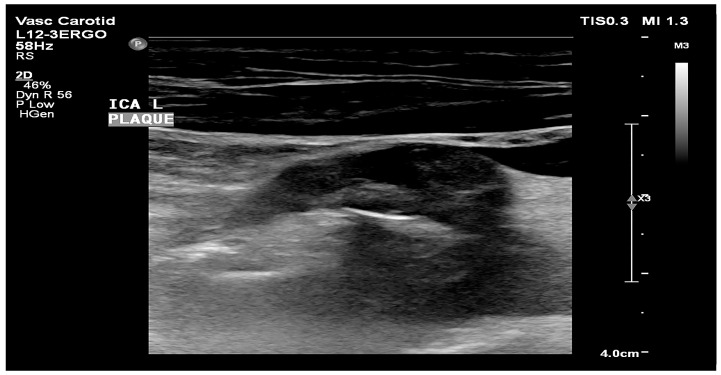
Soft, hypoechoic atherosclerotic plaque in the internal carotid artery (own material).

**Figure 3:**
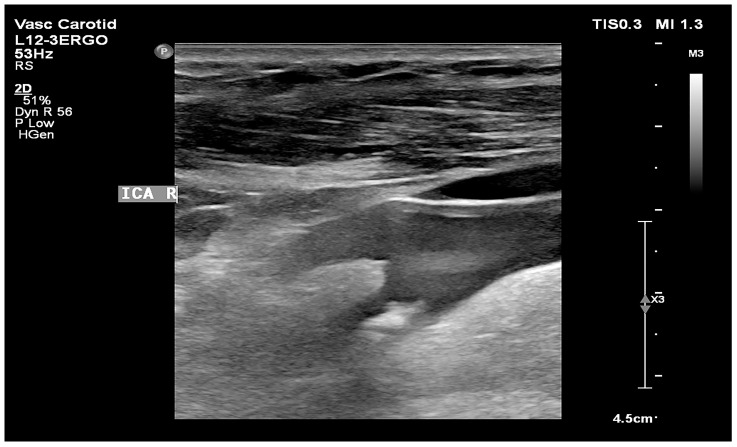
Hard and calcified atherosclerotic plaque in the internal carotid artery. Noteworthy is the significant acoustic shadow (own material).

Low GSM values define the plaque as more hypoechoic and potentially less stable, whereas high GSM values define the plaque as more hyperechoic and potentially more stable [9,10,11,12,19]. An example of unstable plaque with a low GSM value is presented in Figure 2, and stable plaque with a high GSM value is shown in Figure 3.

### 2.4. Statistical Analysis

The normality of the distribution was verified by the Shapiro–Wilk test. Categorical variables are reported as percentages (%), and continuous variables as means and standard deviations (SDs) or medians and interquartile ranges (IQRs). In the statistical analysis, the Chi-square test was used to compare the categorical variables and qualitative features. The Student *t*-test was used for comparisons of normally distributed continuous variables. Comparing quantitative variables without normal distribution, the non-parametric Mann–Whitney U test was used for the two groups. The correlations were analyzed using a linear regression analysis and Spearman’s rank correlation analysis. Multiple regression analyses were performed based on previous results of univariate analyses to assess the combined influence of variables on the GSM scale in each studied group. In this model, the variables with *p* < 0.1 in the univariate analysis were taken into account. The receiver operating characteristic (ROC) curve was used to determine the optimum cutoff GSM value for distinguishing between asymptomatic and symptomatic patients’ group. Statistically significant results were considered at two-tailed *p* < 0.05. Statistica ver 13.1 computer software (StatSoft, Tulsa, OK, USA) was used in the calculations of results. The graphic design presentation of results was prepared using GraphPad Prism 6 (GraphPad Software, La Jolla, CA, USA).

## 3. Results

### 3.1. Patients’ Characteristics

A total of 85 patients with significant ICA stenosis were included in this study—40 of them were symptomatic (having suffered an ischemic stroke, *n* = 28, and/or TIA, *n* = 15, within the past 180 days). Symptomatic patients (after stroke or TIA) were referred to a vascular surgeon by a neurologist, after grouping patients based on their diagnosis, according to the specific type, degree, and extent of CNS ischemia. Patient demographics, comorbidities, and applied pharmacotherapy are presented (Table 1). 

There was no significant difference in the majority of analyzed parameters between symptomatic and asymptomatic groups. There was a higher percentage of male sex and the presence of overweight statuses in the symptomatic than in the asymptomatic group (*p* < 0.05 and *p* < 0.01, respectively). Hypertension was more frequent in the symptomatic than in the asymptomatic group (*p* < 0.01); however, the percentage of patients with grade 3 hypertension was higher in the asymptomatic than in the symptomatic group (*p* < 0.05). The presence of dyslipidemia tended to be also increased in asymptomatic compared to symptomatic patients (*p* = 0.0726). Although similar percentages of patients were treated with hypoglycemic drugs, the sulfonylurea derivatives were taken more often in the asymptomatic than in the symptomatic group (*p* < 0.05). In contrast, the nootropic medication was more often prescribed in the symptomatic than in the asymptomatic group (*p* < 0.01).

### 3.2. GSM Values in Symptomatic and Asymptomatic Group

**Figure 4:**
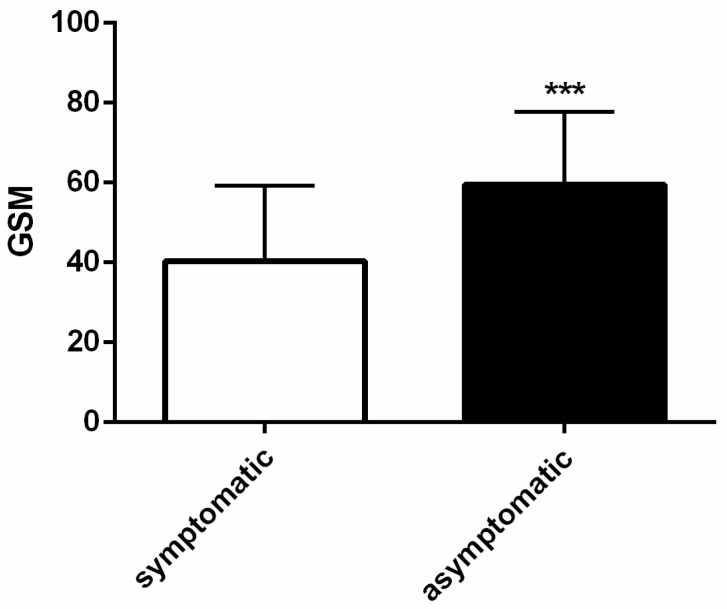
Gray-scale median (GSM) values in symptomatic and asymptomatic patients undergoing carotid endarterectomy. *** *p* < 0.001.

As has been presented in Figure 4, mean GSM in the symptomatic group (40.25 ± 18.99) was significantly lower than in the asymptomatic group (59.41 ± 18.18, *p* < 0.001).

Plaque echogenicity has been categorized into two overarching classes, low and high echogenicity, which reflect lower and higher GSM values. Echolucency is a characteristic indicating the echo-transparency or translucency of an object to ultrasonic waves in sonography. Plaques appearing as echolucent on B-mode ultrasound are primarily lipid-rich, while echogenic plaques are characterized by a higher content of fibrous tissue and calcification. Notably, carotid plaques with an echolucent appearance on ultrasound may signify potential “high-risk” features. Low-echogenic plaques appear as dark, akin to blood, or are predominantly dark (echolucent), while high-echogenic plaques exhibit predominantly bright characteristics, resembling the echo-rich or echogenic properties of the far-wall adventitia interface.

### 3.3. Variables Affecting GSM in Symptomatic and Asymptomatic Group

**Figure 5:**
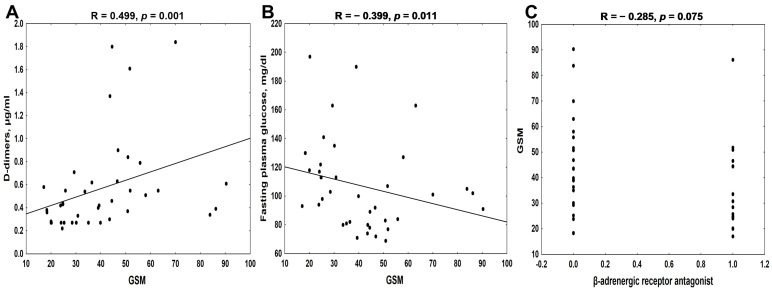
The association between gray-scale median (GSM) values and D-dimers (**A**), fasting plasma glucose (FPG) concentrations (**B**), and β-adrenergic receptor antagonist use (**C**) in symptomatic patients undergoing carotid endarterectomy.

Both in univariate as well as in multiple regression analyses, GSM was significantly associated with D-dimer levels and fasting plasma glucose (FPG) and tended to correlate with β-adrenergic receptor antagonist use in the symptomatic group (Figure 5, Table 2, left side).

**Figure 6:**
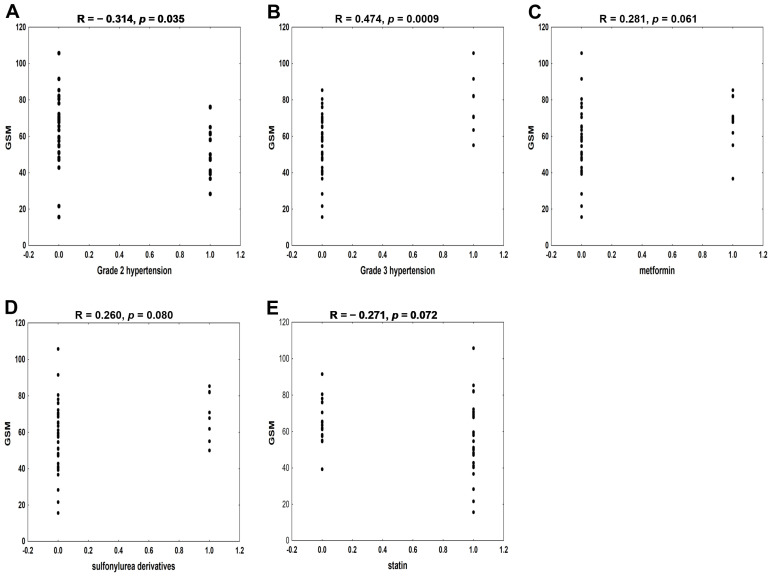
The association between the gray-scale median (GSM) and the presence of grade 2 and 3 hypertension (**A**,**B**), therapy with metformin (**C**), sulfonylurea derivatives (**D**), and statin (**E**) in patients with asymptomatic carotid artery stenosis.

In the group of asymptomatic patients, GSM values were associated with the presence of grade 2 and grade 3 hypertension (*p* < 0.05 and *p* < 0.001; respectively). Moreover, GSM tended to be higher in patients treated with metformin (*p* = 0.061) and sulfonylurea derivatives (*p* = 0.084) compared to subjects untreated with these drugs. Conversely, the GSM scale in the patients taking statin tended to be lower (*p* = 0.071) than in patients who were not treated with statin (Figure 6A–E). In the multiple regression analysis, grade 3 hypertension and statin’s therapy were the independent determinants of GSM in asymptomatic patients (Table 2, right side).

### 3.4. The Ability of GSM to Distinguish Symptomatic from Asymptomatic Patients

**Figure 7:**
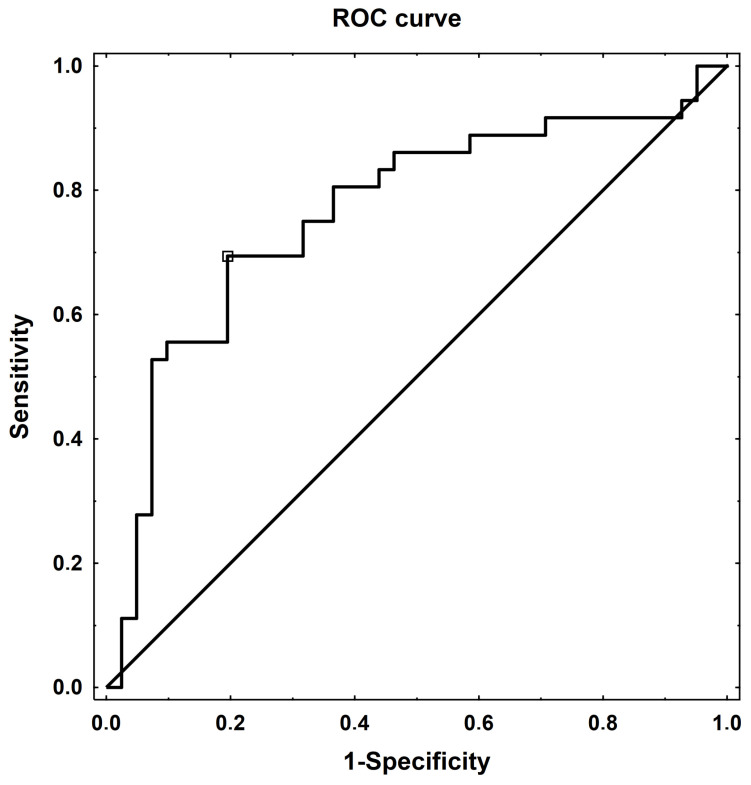
Diagnostic potential of the gray-scale median (GSM) by the receiver operating characteristic (ROC) curve analysis to distinguish patients with symptomatic carotid artery stenosis from those with asymptomatic carotid artery stenosis among all participants. A cutoff point of 46.87 yielded the highest sensitivity and specificity.

The ROC analysis was performed to evaluate the potential ability of GSM to act as a biomarker to discriminate between symptomatic and asymptomatic carotid artery stenosis. As has been shown in Figure 7, the optimal cutoff value was 46.87 with an area under the ROC curve (AUC) of 0.782 (95% CI: 0.679–0.885, *p* < 0.001). The sensitivity and specificity were 73% and 80%, respectively.

## 4. Discussion

In this study, we reported the effect of clinical and demographic parameters, available in routine clinical practice, on the GSM scale in the symptomatic and asymptomatic patients undergoing carotid endarterectomy. The principal finding of this study was that completely diverse clinical parameters affected the GSM values in the symptomatic and asymptomatic patients, whose clinical characteristics were similar in terms of most of the compared parameters (Table 1). Moreover, we demonstrated that the ROC analysis based on GSM may be helpful in differentiating between symptomatic and asymptomatic carotid artery stenosis.

Most previous studies demonstrated that nonmodifiable risk factors, like older age and male sex, are associated with carotid plaque instability [14,21,33]. We did not confirm the effect of age on the GSM scale, probably because the difference in age was not observed between the studied groups. Although a predominance of male sex was observed in the symptomatic group compared to the asymptomatic group, this risk factor was also not related to the GSM scale in any of the studied groups of patients.

In patients with carotid stenosis, both symptomatic and asymptomatic, the analysis of plaque echogenicity allows us to identify patients at high risk of an ischemic cerebral episode [6,7,10,13,19,34]. Our results, according to the previous observation of Grogan et al. [10], showed that carotid plaques of symptomatic patients had lower GSM values than those of asymptomatic patients (Figure 4). Moreover, the nootropic drugs were more often prescribed in the symptomatic than in the asymptomatic group (*p* < 0.01). The use of nootropic drugs is a typical management after an episode of cerebral ischemia, leading to the improvement in the cognitive function and quality of life [35,36,37,38]. Some nootropic drugs, in addition to preventing changes in brain function resulting from hypoxia, can also inhibit platelet aggregation and reduce blood viscosity, preventing future ischemic episodes [38]. 

The statistical analysis performed in the symptomatic group revealed that D-dimers, and fasting plasma glucose (FPG) levels, were associated with GSM values in the univariate analysis (Figure 5), as well as that they were the factors independently affecting this parameter in the multiple regression analysis (Table 2). To our knowledge, this is the first report demonstrating the positive and independent effect of circulating D-dimers on GSM values in symptomatic patients, although there are scarce data indicating the increase in serum D-dimers in patients with carotid atherosclerotic stenosis [39,40], especially in those with unstable plaque [41]. Krupinski et al. [41] demonstrated that although plasma and plaque D-dimers were associated with unstable carotid disease, there was no association between them, indicating that circulating D-dimers did not reflect intraplaque D-dimer generation but rather were the marker of the general procoagulant state. Thus, one would rather expect D-dimers to be inversely correlated with GSM values. From a clinical perspective, serum D-dimers represent the extent of the total thrombolytic activity in the body, and their elevated levels are a direct consequence of fibrinolysis, being a result of the earlier activation of the coagulation system [42]. Houard et al. [43] observed that the thrombus was the epicenter of fibrinolytic activity within mural thrombi of human abdominal aortic aneurysms, leading to the release of D-dimers into the blood. If this scenario might exist in other clinical situations involving the activation of the coagulation/fibrinolysis system, it seems to be possible that, observed in the present study, independent and positive impact of D-dimers on the GSM scale can reflect a protective effect of the fibrinolytic system against procoagulant intravascular activity, promoting by this way more stable carotid plaque. On the other hand, in the only study performed in patients with stable CAD, Kothari et al. [44] showed that plasma D-dimers were positively associated with coronary artery plaque calcium, and inversely associated with plaque fibrosis. Moreover, increased values of D-dimers (>500 ng/mL) were also positively correlated with plaque necrosis in this study. As the large necrotic core and thin fibrous cap are characteristic features of unstable plaque [8,9,10,11,12], the observed-by-us positive relationship between circulating D-dimers and GSM values could explain the link between fibrin degradation, carotid plaque calcification, and symptoms of unstable carotid atherosclerosis in the group of symptomatic patients. This is also in agreement with results of Kan et al. [45], who demonstrated that the calcification of carotid plaque may be a risk factor for stroke.

In the symptomatic group, mean GSM was significantly correlated with fasting plasma glucose (FPG) levels (Figure 5B), which remained an independent determinant of the GSM scale (Table 2). Despite the lack of difference in diabetes prevalence and FPG levels between symptomatic and asymptomatic groups, the lower FPG values correlated with a higher GSM scale in the former group. This is in agreement with previous studies, in which the association between plaque instability and the level of glycemia has been established [15,46,47]. Huang et al. [48] observed that soft plaques are more present in diabetic patients and that the higher levels of glycated hemoglobin (HbA1c) are associated with unstable plaques in ultrasonic images. Moreover, the echogenicity of the intima–media complex in the common carotid artery is independently related to insulin resistance in elderly men [49]. These data indicate that hyperglycemia may contribute to the creation of echolucent plaque, which can be reverted by adequate glycemic control.

The therapy with β-adrenergic receptor antagonists tended to correlate with lower values of the GSM scale (Figure 5C, Table 2) in symptomatic patients. This is in contrast to the previous studies, demonstrating that the use of β-blockers was associated with higher GSM values of carotid plaque [50,51]. However, the results of two randomized, ultrasound studies, the Beta-Blocker Cholesterol Lowering Asymptomatic Plaque Study (BCAPS) [52] and the Effect of Long-Term Treatment of Metoprolol CR/XL on Surrogate Variables for Atherosclerotic Disease (ELVA) study [53], revealed the favorable effects of β -blockers on early stages of atherosclerosis development, yet without symptoms of carotid artery disease. Our symptomatic patients were more likely to have chronic illnesses, like hypertension (97.5% vs. 77.8%) and overweight status (55 vs. 29%), compared with asymptomatic ones. The use of antihypertensive drugs (90% vs. 80%), and especially RAAS inhibitors (91.7% vs. 81.1%), was also slightly higher in the symptomatic group than in asymptomatic patients. This suggests that symptomatic patients presented more severe changes in the cardiovascular system, which required more intensive pharmacotherapy. In our opinion, this might explain the inverse association between β-blocker use and the GSM scale in our symptomatic patients.

The completely different factors influenced the GSM values in the asymptomatic group of patients undergoing the CEA procedure. In this group, the degree of hypertension and use of hypoglycemic and lipid-lowering pharmacotherapy were associated (or tended to associate) with GSM values in the univariate analysis (Figure 6). However, only statin therapy and grade 3 hypertension turned out to be independent factors affecting GSM in this group. It is well documented that chronic statin treatment slows atherosclerosis progression, favors carotid plaque stabilization, and reduces the risk of stroke through a plethora of different mechanisms, as reviewed deeply by Paraskevas et al. [54]. From this perspective, the statin therapy should stabilize the carotid plaque, which would increase GSM values. However, the statin users in our study tended to have lower GSM values than non-users (Figure 6E), and statin therapy was one of the factors independently affecting the GSM scale in asymptomatic patients (Table 2). It is now believed that statins may exert a dual action in relations to atherosclerotic plaque. On the one hand, in advanced, established plaque, the statins’ treatment can increase the calcification [55,56]. On the other hand, in developing plaques, statins can slow down (or even inhibit) atherogenic mechanisms, such as calcium deposition [57], and our results appear to be in keeping with this latter opinion.

Hypertension is the most common risk factor for ischemic stroke, and the effective lowering of blood pressure can significantly reduce this risk [58,59]. Although hypertension was significantly more frequent in our symptomatic patients than in asymptomatic patients (*p* < 0.01), the grade 3 hypertension was more common in the asymptomatic group. In this latter group, we noticed the separate association between the grade of hypertension and GSM; namely, the presence of grade 2 hypertension was inversely associated with GSM values, indicating the typical relation of hypertension with a more vulnerable plaque phenotype [58,59]. In contradiction to this, the grade 3 hypertension independently and positively affected the GSM scale, suggesting the association of severe hypertension with more stable carotid plaque. We observed that patients with the grade 3 hypertension were treated with fixed triple/quadruple combinations of antihypertensive drugs, including an RAAS inhibitor in combination with a β-blocker, calcium channel antagonist, and/or diuretic. Moreover, most of them were also taking statins and aspirin. Such pharmacotherapy has been reported to have a hyperadditive anti-inflammatory effect [60,61] and may slow down atherosclerotic plaque progression and prevent their destabilization [62,63]. It is therefore possible that, despite significant hypertension, these patients remained asymptomatic for a long time, whereas their atherosclerotic plaques tended to harden and calcify over time.

The GSM scale tended to be higher in asymptomatic patients treated with oral hypoglycemic drugs, particularly with metformin (Figure 6C) and sulfonylurea derivatives, compared to subjects untreated with these drugs. This is in agreement with results of previous studies, which suggest the beneficial effect of these hypoglycemic drugs on atherosclerotic plaque stabilization, independently of their antidiabetic effect [64,65]. However, in the multivariable analysis, these antidiabetic drugs no longer affected the GSM scale in our asymptomatic patients.

To identify asymptomatic and symptomatic patients with greater accuracy on the basis of GSM values, the ROC analysis was performed. The optimum cutoff GSM value that would best distinguish symptomatic from asymptomatic carotid artery stenosis was 46.87, with sensitivity of 73% and specificity of 80% (Figure 7). These results indicate that GSM showed a high discriminative ability and could be useful in clinical practice. 

### Study Limitations

A potential limitation of our study is its cross-sectional nature and the inclusion of well-documented atherosclerotic risk factors, whereas other factors of possible importance for atherosclerosis, for example, diet, were not considered. Another limitation is the requisite utilization of specialized software for post-processing of pre-existing ultrasound images, a processing that is tedious and each time requires manual marking of the examined objects and reference areas, as well as the inability to directly compare the ultrasound image with the histopathological image of the plaque. However, the prior analyses have already established a remarkably high degree of correlation between the ultrasound depiction of atherosclerotic plaque and its histopathological characteristics [8,9,10,11,12]. Consequently, we chose not to undertake histopathological examinations based on this existing substantial correlation.

## 5. Conclusions

The results of the present study indicated that the completely diverse clinical parameters may affect the GSM values in the symptomatic and asymptomatic patients undergoing the CEA procedure. D-dimers and fasting plasma glucose levels were independently associated with GSM values in the symptomatic group, whereas statins’ therapy and grade 3 hypertension independently affected this parameter in asymptomatic patients. For clinical practice, distinguishing plaques that may cause clinical symptoms from those that are asymptomatic is very important for management of patients with ICA stenosis. It is important to highlight that patients, particularly those within the 51–70% stenosis range, demand a personalized approach, and the decision for surgical intervention is not straightforward. In many clinical settings, the decision to revascularize such patients is often made subjectively. Meanwhile, the qualification of symptomatic patients for interventional treatment is recommended, and such a decision in the case of asymptomatic patients is not easy, because it carries the risk of complications. Moreover, the optimized medical treatment was shown to significantly reduce the risk of stroke among patients with asymptomatic ICA stenosis, and seems to be as effective as CEA for this group of patients [66]. Our method seeks to reduce this subjectivity by adopting a comprehensive approach that takes into account individual risk factors and the overall patient’s profile. Unlike methods that focus solely on the severity of stenosis, our approach considers both the morphology and stability of the atherosclerotic plaque. The identification of GSM as a clinically useful parameter for differentiating between symptomatic and asymptomatic ICA stenosis, as well as establishing the commonly available clinical parameters, which can impact the echogenicity of the atherosclerotic plaque, will allow for expanding the knowledge about cerebrovascular risk, which would be of great importance for the treatment and selection of high-risk patients. We believe that our modern, objective, and holistic method—which accounts for all patient characteristics and the specific attributes of the atherosclerotic plaque—will enhance stroke prevention and reduce mortality. By incorporating a comprehensive evaluation, our approach promises more effective decision-making and improved outcomes for patients with carotid stenosis.

## Figures and Tables

**Table 1:** Clinical and demographical characteristics of symptomatic and asymptomatic patients undergoing carotid endarterectomy.

	Symptomatic Patients (*n* = 40)	Asymptomatic Patients (*n* = 45)	*p*
Age, years	68.57 ± 8.29	67.91 ± 8.52	NS
Sex, male (%)	35 (87.5)	31 (68.9)	0.0175
Body mass index, kg/m^2^	27.24 ± 3.33	26.94 ± 4.98	NS
Overweight, *n* (%)	22 (55)	13 (29)	0.0080
Obesity, *n* (%)	9 (22.5)	11 (24.4)	NS
Cardiovascular disease, *n* (%)	9 (22.5)	11 (24.4)	NS
Peripheral artery disease, *n* (%)	18 (45)	19 (42.2)	NS
Chronic kidney disease, *n* (%)	5 (12.5)	8 (17.8)	NS
Hypertension, *n* (%)	39 (97.5)	35 (77.8)	0.0028
Grade 1 hypertension, *n* (%)	15 (38.5)	12 (34.3)	NS
Grade 2 hypertension, *n* (%)	21 (53.8)	16 (45.7)	NS
Grade 3 hypertension, *n* (%)	3 (7.7)	8 (22.9)	0.036
Diabetes, *n* (%)	13 (32.5)	17 (37.8)	NS
Smoking, *n* (%)	33 (82.5)	33 (73.3)	NS
Packs per year	38 (15–50)	30 (20–45)	NS
Dyslipidemia, *n* (%)	23 (57.5)	33 (73.3)	0.0726
hsCRP, mg/L	2.5 (0.9–4.4)	3.1 (1.5–7.8)	NS
D-dimers, µg/mL	0.43 (0.29–0.62)	0.37 (0.28–0.72)	NS
Fasting plasma glucose, mg/dL	101.5 (82.5–120)	92 (82–113)	NS
Pharmacotherapy:			
Hypoglycemic drugs, *n* (%)	11 (27.5)	14 (31.1)	NS
Sulfonylurea derivatives, *n* (%)	2 (18.2)	8 (57.1)	0.024
Metformin, *n* (%)	9 (81.8)	11 (78.6)	NS
Antihypertensive drugs, *n* (%)	36 (90)	37 (82)	NS
Diuretics, *n* (%)	13 (36.1)	16 (43.2)	NS
β-adrenergic receptor antagonist, *n* (%)	18 (50)	21 (56.8)	NS
Calcium channel antagonist, *n* (%)	14 (38.9)	16 (43.2)	NS
RAAS inhibitor, *n* (%)	33 (91.7)	30 (81.1)	NS
Antiplatelet drugs, *n* (%)	29 (72.5)	32 (71.1)	NS
Statins, *n* (%)	27 (67.5)	29 (64.4)	NS
Nootropic drugs, *n* (%)	11 (27.5)	5 (11.11)	0.0043

Data are means ± SD or median and interquartile ranges; *n*—number of patients; NS–not statistically significant; hsCRP—high-sensitivity C-reactive protein; RAAS—renin–angiotensin–aldosterone system.

**Table 2:** Variables predicted gray-scale median (GSM) in multiple regression analysis in symptomatic and asymptomatic patients.

Symptomatic Group				Asymptomatic Group *			
Independent Variable	Regression Coefficient	Standard Error	*p* Value	Independent Variable	Regression Coefficient	Standard Error	*p* Value
D-dimers	0.359	0.153	0.025	Statin	−0.299	0.135	0.033
Fasting plasma glucose	−0.345	0.156	0.034	Grade 3 hypertension	0.302	0.142	0.042
Β-adrenergic receptor antagonist	−0.284	0.148	0.065				

Multiple r for variables in the model = 0.658 (* 0.690), adjusted r^2^ = 0.330 (* 0.350), *p* < 0.01 (* <0.001).

## Data Availability

The data presented in this study are available on request from the corresponding author. All requests will be checked according to privacy and possible ethical restrictions.

## References

[1] Johnson W., Onuma O., Owolabi M., Sachdev S. (2016). Stroke: A Global Response Is Needed. Bull. World Health Organ..

[2] Ooi Y.C., Gonzalez N.R. (2015). Management of Extracranial Carotid Artery Disease. Cardiol. Clin..

[3] Brinjikji W., Huston J., Rabinstein A.A., Kim G.-M., Lerman A., Lanzino G. (2016). Contemporary Carotid Imaging: From Degree of Stenosis to Plaque Vulnerability. J. Neurosurg..

[4] Migdalski A., Jawien A. (2021). New Insight into Biology, Molecular Diagnostics and Treatment Options of Unstable Carotid Atherosclerotic Plaque: A Narrative Review. Ann. Transl. Med..

[5] Golemati S., Gastounioti A., Nikita K.S. (2013). Toward Novel Noninvasive and Low-Cost Markers for Predicting Strokes in Asymptomatic Carotid Atherosclerosis: The Role of Ultrasound Image Analysis. IEEE Trans. Biomed. Eng..

[6] Grønholdt M.-L.M., Nordestgaard B.G., Schroeder T.V., Vorstrup S., Sillesen H. (2001). Ultrasonic Echolucent Carotid Plaques Predict Future Strokes. Circulation.

[7] Biasi G.M., Sampaolo A., Mingazzini P., De Amicis P., El-Barghouty N., Nicolaides A.N. (1999). Computer Analysis of Ultrasonic Plaque Echolucency in Identifying High Risk Carotid Bifurcation Lesions. Eur. J. Vasc. Endovasc. Surg..

[8] Spanos K., Tzorbatzoglou I., Lazari P., Maras D., Giannoukas A.D. (2018). Carotid Artery Plaque Echomorphology and Its Association with Histopathologic Characteristics. J. Vasc. Surg..

[9] Doonan R.J., Gorgui J., Veinot J.P., Lai C., Kyriacou E., Corriveau M.M., Steinmetz O.K., Daskalopoulou S.S. (2016). Plaque Echodensity and Textural Features Are Associated with Histologic Carotid Plaque Instability. J. Vasc. Surg..

[10] Grogan J.K., Shaalan W.E., Cheng H., Gewertz B., Desai T., Schwarze G., Glagov S., Lozanski L., Griffin A., Castilla M. (2005). B-Mode Ultrasonographic Characterization of Carotid Atherosclerotic Plaques in Symptomatic and Asymptomatic Patients. J. Vasc. Surg..

[11] Mitchell C.C., Stein J.H., Cook T.D., Salamat S., Wang X., Varghese T., Jackson D.C., Sandoval Garcia C., Wilbrand S.M., Dempsey R.J. (2017). Histopathologic Validation of Grayscale Carotid Plaque Characteristics Related to Plaque Vulnerability. Ultrasound Med. Biol..

[12] Salem M.K., Bown M.J., Sayers R.D., West K., Moore D., Nicolaides A., Robinson T.G., Naylor A.R. (2014). Identification of Patients with a Histologically Unstable Carotid Plaque Using Ultrasonic Plaque Image Analysis. Eur. J. Vasc. Endovasc. Surg..

[13] Madycki G., Staszkiewicz W., Gabrusiewicz A. (2006). Carotid Plaque Texture Analysis Can Predict the Incidence of Silent Brain Infarcts Among Patients Undergoing Carotid Endarterectomy. Eur. J. Vasc. Endovasc. Surg..

[14] Karim R., Xu W., Kono N., Li Y., Yan M., Stanczyk F.Z., Hodis H.N., Mack W.J. (2023). Comparison of Cardiovascular Disease Risk Factors Between 2 Subclinical Atherosclerosis Measures in Healthy Postmenopausal Women: Carotid Artery Wall Thickness and Echogenicity. J. Ultrasound Med..

[15] Della-Morte D., Dong C., Crisby M., Gardener H., Cabral D., Elkind M.S.V., Gutierrez J., Sacco R.L., Rundek T. (2022). Association of Carotid Plaque Morphology and Glycemic and Lipid Parameters in the Northern Manhattan Study. Front. Cardiovasc. Med..

[16] Mastroiacovo D., Mengozzi A., Dentali F., Pomero F., Virdis A., Camerota A., Muselli M., Necozione S., Bocale R., Ferri C. (2023). Enhanced Carotid Plaque Echolucency Is Associated with Reduced Cognitive Performance in Elderly Patients with Atherosclerotic Disease Independently on Metabolic Profile. Metabolites.

[17] Coggi D., Frigerio B., Bonomi A., Ruscica M., Ferri N., Sansaro D., Ravani A., Ferrante P., Damigella M., Veglia F. (2021). Relationship between Circulating PCSK9 and Markers of Subclinical Atherosclerosis—The IMPROVE Study. Biomedicines.

[18] Konishi T., Funayama N., Yamamoto T., Morita T., Hotta D., Nomura R., Nakagaki Y., Murahashi T., Kamiyama K., Yoshimoto T. (2018). Pathological Quantification of Carotid Artery Plaque Instability in Patients Undergoing Carotid Endarterectomy. Circ. J..

[19] Ariyoshi K., Okuya S., Kunitsugu I., Matsunaga K., Nagao Y., Nomiyama R., Takeda K., Tanizawa Y. (2015). Ultrasound Analysis of Gray-scale Median Value of Carotid Plaques Is a Useful Reference Index for Cerebro-cardiovascular Events in Patients with Type 2 Diabetes. J. Diabetes Investig..

[20] Anbar R., Chaturvedi N., Eastwood S.V., Tillin T., Hughes A.D. (2023). Carotid Atherosclerosis in People of European, South Asian and African Caribbean Ethnicity in the Southall and Brent Revisited Study (SABRE). Front. Cardiovasc. Med..

[21] Puz P., Urbanek T., Ziaja D., Cieślik A., Stęposz A., Lasek-Bal A. (2020). Factors Associated with the Symptomatic Status of Carotid Artery Stenosis: Identification in a Cross-Sectional Study and Development of a Scoring System. Pol. Arch. Intern. Med..

[22] Ferguson G.G., Eliasziw M., Barr H.W.K., Clagett G.P., Barnes R.W., Wallace M.C., Taylor D.W., Haynes R.B., Finan J.W., Hachinski V.C. (1999). The North American Symptomatic Carotid Endarterectomy Trial: Surgical Results in 1415 Patients. Stroke.

[23] Bonati L.H., Kakkos S., Berkefeld J., De Borst G.J., Bulbulia R., Halliday A., Van Herzeele I., Koncar I., McCabe D.J., Lal A. (2021). European Stroke Organisation Guideline on Endarterectomy and Stenting for Carotid Artery Stenosis. Eur. Stroke J..

[24] Araszkiewicz A., Bandurska-Stankiewicz E., Borys S., Budzyński A., Cyganek K., Cypryk K., Czech A., Czupryniak L., Drzewoski J., Dzida G. (2022). 2022 Guidelines on the Management of Patients with Diabetes. A Position of Diabetes Poland. Curr. Top Diabetes.

[25] Solnica B., Sygitowicz G., Sitkiewicz D., Cybulska B., Jóźwiak J., Odrowąż-Sypniewska G., Banach M. (2020). 2020 Guidelines of the Polish Society of Laboratory Diagnostics (PSLD) and the Polish Lipid Association (PoLA) on Laboratory Diagnostics of Lipid Metabolism Disorders. Arch. Med. Sci..

[26] Knuuti J., Wijns W., Saraste A., Capodanno D., Barbato E., Funck-Brentano C., Prescott E., Storey R.F., Deaton C., Cuisset T. (2020). 2019 ESC Guidelines for the Diagnosis and Management of Chronic Coronary Syndromes. Eur. Heart J..

[27] Levey A.S., Stevens L.A., Schmid C.H., Zhang Y., Castro A.F., Feldman H.I., Kusek J.W., Eggers P., Van Lente F., Greene T. (2009). A New Equation to Estimate Glomerular Filtration Rate. Ann. Intern. Med..

[28] Inker L.A., Astor B.C., Fox C.H., Isakova T., Lash J.P., Peralta C.A., Kurella Tamura M., Feldman H.I. (2014). KDOQI US Commentary on the 2012 KDIGO Clinical Practice Guideline for the Evaluation and Management of CKD. Am. J. Kidney Dis..

[29] Bermúdez-López M., Martí-Antonio M., Castro-Boqué E., Bretones M.D.M., Farràs C., Gonzalez J., Pamplona R., Lecube A., Mauricio D., Cambray S. (2023). Cumulative Tobacco Consumption Has a Dose-Dependent Effect on Atheromatosis Burden and Improves Severe Atheromatosis Prediction in Asymptomatic Middle-Aged Individuals: The ILERVAS Study. Atherosclerosis.

[30] Williams B., Mancia G., Spiering W., Agabiti Rosei E., Azizi M., Burnier M., Clement D.L., Coca A., De Simone G., Dominiczak A. (2018). 2018 ESC/ESH Guidelines for the Management of Arterial Hypertension. Eur. Heart J..

[31] Yumuk V., Tsigos C., Fried M., Schindler K., Busetto L., Micic D., Toplak H. (2015). European Guidelines for Obesity Management in Adults. Obes. Facts..

[32] Kaszczewski P., Elwertowski M., Leszczyński J., Ostrowski T., Gałązka Z. (2022). Volumetric Flow Assessment in Doppler Ultrasonography in Risk Stratification of Patients with Internal Carotid Stenosis and Occlusion. J. Clin. Med..

[33] Catalan M., Herreras Z., Pinyol M., Sala-Vila A., Amor A.J., De Groot E., Gilabert R., Ros E., Ortega E. (2015). Prevalence by Sex of Preclinical Carotid Atherosclerosis in Newly Diagnosed Type 2 Diabetes. Nutr. Metab. Cardiovasc. Dis..

[34] Howard D.P.J., Van Lammeren G.W., Rothwell P.M., Redgrave J.N., Moll F.L., De Vries J.-P.P.M., De Kleijn D.P.V., Den Ruijter H.M., De Borst G.J., Pasterkamp G. (2015). Symptomatic Carotid Atherosclerotic Disease: Correlations Between Plaque Composition and Ipsilateral Stroke Risk. Stroke.

[35] Lal B.K., Dux M.C., Sikdar S., Goldstein C., Khan A.A., Yokemick J., Zhao L. (2017). Asymptomatic Carotid Stenosis Is Associated with Cognitive Impairment. J. Vasc. Surg..

[36] Lazar R.M., Wadley V.G., Myers T., Jones M.R., Heck D.V., Clark W.M., Marshall R.S., Howard V.J., Voeks J.H., Manly J.J. (2021). Baseline Cognitive Impairment in Patients With Asymptomatic Carotid Stenosis in the CREST-2 Trial. Stroke.

[37] Zongfang Z., Wenjing L., Zhaomin C., Lei Z. (2020). Therapeutic Effect of Piracetam with Nimodipine on Vascular Dementia after Cerebral Infarction. Pak. J. Pharm. Sci..

[38] Teng C.M., Yeh H.I., Lee L.G. (1983). Anticoagulant and Antiplatelet Properties of Piracetam. Taiwan. Yi Xue Hui Za Zhi..

[39] Kong Y., Kong Y., Dai Y., Zhang J. (2021). Prognostic Value of Color Doppler Ultrasound, D-Dimer, and Lp-PLA2 Levels in Carotid Atherosclerotic Stenosis. Am. J. Transl. Res..

[40] Wang J., Huang R., Tian S., Lin H., Guo D., An K., Wang S. (2019). Elevated Plasma Level of D-Dimer Predicts the High Risk of Early Cognitive Impairment in Type 2 Diabetic Patients as Carotid Artery Plaques Become Vulnerable or Get Aggravated. Curr. Alzheimer Res..

[41] Krupinski J., Catena E., Miguel M., Domenech P., Vila R., Morchon S., Rubio F., Cairols M., Slevin M., Badimon L. (2007). D-Dimer Local Expression Is Increased in Symptomatic Patients Undergoing Carotid Endarterectomy. Int. J. Cardiol..

[42] Bruinstroop E., Van De Ree M.A., Huisman M.V. (2009). The Use of D-Dimer in Specific Clinical Conditions: A Narrative Review. Eur. J. Intern. Med..

[43] Houard X., Rouzet F., Touat Z., Philippe M., Dominguez M., Fontaine V., Sarda-Mantel L., Meulemans A., Le Guludec D., Meilhac O. (2007). Topology of the Fibrinolytic System within the Mural Thrombus of Human Abdominal Aortic Aneurysms. J. Pathol..

[44] Kothari H., Nguyen A.T., Yang X., Hisada Y., Tsimikas S., Mackman N., Taylor A., McNamara C.A. (2018). Association of D-Dimer with Plaque Characteristics and Plasma Biomarkers of Oxidation-Specific Epitopes in Stable Subjects with Coronary Artery Disease. J. Cardiovasc. Transl. Res..

[45] Kan Y., He W., Ning B., Li H., Wei S., Yu T. (2019). The Correlation between Calcification in Carotid Plaque and Stroke: Calcification May Be a Risk Factor for Stroke. Int. J. Clin. Exp. Pathol..

[46] Huang J., Jiao S., Song Y., Chen Y., Zhang J., Zhang C., Gong T., Chen M. (2020). Association between Type 2 Diabetes Mellitus, Especially Recently Uncontrolled Glycemia, and Intracranial Plaque Characteristics: A High-resolution Magnetic Resonance Imaging Study. J. Diabetes Investig..

[47] Genkel V.V., Kuznetsova A.S., Lebedev E.V., Shaposhnik I.I. (2021). Factors Associated with Atherosclerotic Plaque Echogenicity in Patients Aged 40-64 with Carotid Atherosclerosis. Kardiologiia.

[48] Huang X.-W., Zhang Y.-L., Meng L., Qian M., Zhou W., Zheng R.-Q., Zheng H.-R., Niu L. (2016). The Relationship between HbA1c and Ultrasound Plaque Textures in Atherosclerotic Patients. Cardiovasc. Diabetol..

[49] Lind L., Wohlin M., Andren B., Sundström J. (2013). The Echogenicity of the Intima–Media Complex in the Common Carotid Artery Is Related to Insulin Resistance Measured by the Hyperinsulinemic Clamp in Elderly Men. Clin. Physiol. Funct. Imaging..

[50] Asciutto G., Dias N.V., Persson A., Nilsson J., Gonçalves I. (2014). Treatment with Betablockers Is Associated with Higher Grey-Scale Median in Carotid Plaques. BMC Cardiovasc. Disord..

[51] Östling G., Gonçalves I., Wikstrand J., Berglund G., Nilsson J., Hedblad B. (2011). Long-Term Treatment with Low-Dose Metoprolol CR/XL Is Associated with Increased Plaque Echogenicity: The Beta-Blocker Cholesterol-Lowering Asymptomatic Plaque Study (BCAPS). Atherosclerosis.

[52] Hedblad B., Wikstrand J., Janzon L., Wedel H., Berglund G. (2001). Low-Dose Metoprolol CR/XL and Fluvastatin Slow Progression of Carotid Intima-Media Thickness: Main Results From the β-Blocker Cholesterol-Lowering Asymptomatic Plaque Study (BCAPS). Circulation.

[53] Wiklund O., Hulthe J., Wikstrand J., Schmidt C., Olofsson S.-O., Bondjers G. (2002). Effect of Controlled Release/Extended Release Metoprolol on Carotid Intima-Media Thickness in Patients With Hypercholesterolemia: A 3-Year Randomized Study. Stroke.

[54] Paraskevas K.I., Gloviczki P., Antignani P.L., Comerota A.J., Dardik A., Davies A.H., Eckstein H.-H., Faggioli G., Fernandes E., Fernandes J. (2022). Benefits and Drawbacks of Statins and Non-Statin Lipid Lowering Agents in Carotid Artery Disease. Prog. Cardiovasc. Dis..

[55] Xian J.Z., Lu M., Fong F., Qiao R., Patel N.R., Abeydeera D., Iriana S., Demer L.L., Tintut Y. (2021). Statin Effects on Vascular Calcification: Microarchitectural Changes in Aortic Calcium Deposits in Aged Hyperlipidemic Mice. Arterioscler. Thromb. Vasc. Biol..

[56] Osei A.D., Mirbolouk M., Berman D., Budoff M.J., Miedema M.D., Rozanski A., Rumberger J.A., Shaw L., Al Rifai M., Dzaye O. (2021). Prognostic Value of Coronary Artery Calcium Score, Area, and Density among Individuals on Statin Therapy vs. Non-Users: The Coronary Artery Calcium Consortium. Atherosclerosis.

[57] Shioi A., Ikari Y. (2018). Plaque Calcification During Atherosclerosis Progression and Regression. J. Atheroscler. Thromb..

[58] Marfella R., Siniscalchi M., Portoghese M., Di Filippo C., Ferraraccio F., Schiattarella C., Crescenzi B., Sangiuolo P., Ferraro G., Siciliano S. (2007). Morning Blood Pressure Surge as a Destabilizing Factor of Atherosclerotic Plaque: Role of Ubiquitin–Proteasome Activity. Hypertension.

[59] Fassaert L.M.M., Timmerman N., Van Koeverden I.D., Pasterkamp G., De Kleijn D.P.V., De Borst G.J. (2019). Preoperative Hypertension Is Associated with Atherosclerotic Intraplaque Hemorrhage in Patients Undergoing Carotid Endarterectomy. Atherosclerosis.

[60] Paraskevas K.I., Cambria R.P. (2018). Best Medical Treatment for Patients with Carotid Stenosis: Evidence-Based Medicine or Wishful Thinking?. Angiology.

[61] Morris T., Stables M., Hobbs A., De Souza P., Colville-Nash P., Warner T., Newson J., Bellingan G., Gilroy D.W. (2009). Effects of Low-Dose Aspirin on Acute Inflammatory Responses in Humans. J. Immunol..

[62] Artom N., Montecucco F., Dallegri F., Pende A. (2014). Carotid Atherosclerotic Plaque Stenosis: The Stabilizing Role of Statins. Eur. J. Clin. Investig..

[63] Zhang L., Li Z., Wu Y., Fan Y., He Z., He P., Liang J. (2022). Cinnamon and Aspirin for Mild Ischemic Stroke or Transient Ischemic Attack: A Pilot Trial. Clin. Ther..

[64] Kataoka Y., Nicholls S.J., Andrews J., Uno K., Kapadia S.R., Tuzcu E.M., Nissen S.E., Puri R. (2022). Plaque Microstructures during Metformin Therapy in Type 2 Diabetic Subjects with Coronary Artery Disease: Optical Coherence Tomography Analysis. Cardiovasc. Diagn. Ther..

[65] Renier G., Mamputu J.-C., Serri O. (2003). Benefits of Gliclazide in the Atherosclerotic Process: Decrease in Monocyte Adhesion to Endothelial Cells. Metabolism.

[66] Spence J.D., Song H., Cheng G. (2016). Appropriate Management of Asymptomatic Carotid Stenosis. Stroke Vasc. Neurol..

